# Genome-Wide miRNA Seeds Prediction in Archaea

**DOI:** 10.1155/2014/671059

**Published:** 2014-05-14

**Authors:** Shengqin Wang, Yuming Xu, Zuhong Lu

**Affiliations:** ^1^State Key Lab of Bioelectronics, School of Biological Science and Medical Engineering, Southeast University, Nanjing 210096, China; ^2^Department of Biomedical Engineering, College of Engineering, Peking University, Beijing 100781, China

## Abstract

Growing evidence indicates that miRNA genes exist in the archaeal genome, though the functional role of such noncoding RNA remains unclear. Here, we integrated the phylogenetic information of available archaeal genomes to predict miRNA seeds (typically defined as the 2–8 nucleotides of mature miRNAs) on the genomic scale. Finally, we found 2649 candidate seeds with significant conservation signal. Eleven of 29 unique seeds from previous study support our result (*P* value <0.01), which demonstrates that the pipeline is suitable to predict experimentally detectable miRNA seeds. The statistical significance of the overlap between the detected archaeal seeds and known eukaryotic seeds shows that the miRNA may evolve before the divergence of these two domains of cellular life. In addition, miRNA targets are enriched for genes involved in transcriptional regulation, which is consistent with the situation in eukaryote. Our research will enhance the regulatory network analysis in Archaea.

## 1. Introduction

Archaea, which is similar to bacteria in morphology [1] yet more in common with eukaryote in genetic features [2], represents unique molecular characteristics on genomic level [3]. As a life form thriving in an extreme environment, Archaea's regulatory network remains unclear.

miRNAs are important regulators of gene expression within a complex regulatory network, which mediate the inhibition of translation or trigger degradation by paring with target mRNAs in posttranslational regulation to control gene expression [4]. Though the exact mechanism is still unknown, many studies support that miRNAs play a significant role in Archaea. For example, there exist some important miRNAs related functional elements in archaeal genome, such as eukaryotic RNA silencing pathway key protein [5, 6], the conserved domain sequence of Dicer [6], and the abundant circular RNAs which perform as the efficient miRNA sponges in animals [7–9]. Recently, with the development of high-throughput sequencing and comprehensive transcriptome analysis techniques, a number of miRNAs have been detected in Archaea [10].

For such kind of life living in an extreme environment, the experimental testing and verification of miRNAs and their function are costly, labor intensive, and vulnerable to environmental conditions. Here, we integrated the evidence of phylogenetic information in different species to identify conserved miRNA seeds on a genome-wide scale, to optimize the selection of putative miRNA and their targets for subsequent experimental investigation.

## 2. Material and Methods

Data of complete genome sequences, “Genbank Refseq” gene annotations, and conservation information of each position were downloaded from the UCSC Archaeal Table Browser [11]. The data included 115 genomes, which covers all the four recognized phyla up to date. Plasmids and unplaced scaffolds were excluded for further analysis.

**Figure 1:**
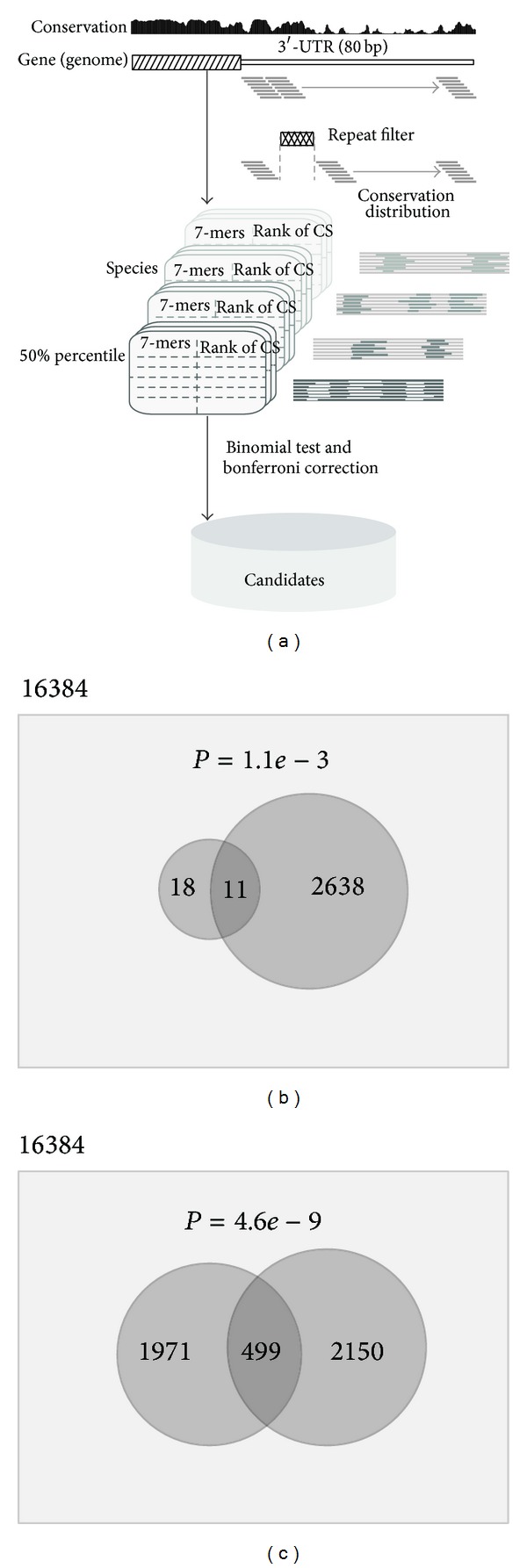
(a) Graphical representation of the miRNA seed prediction pipeline. Based on the defined 3′-UTR from “Genbank Refseq” gene annotation, in genome sequences, we employed a sliding window method that used continuous 7 nt window with a step size of 1 nt starting from the 5′ terminal of defined 3′-UTR and calculated the WCS by the average conservation score in each window. We discarded sliding windows overlapped with repeat sequence. Then, we ranked all of these WCSs within each species and calculated the counts of conservation scores greater or less than the score of CS50, respectively. At last, for each type of 7-mers, we compared these two kinds of counts in all species to check whether the difference between them is significant using the binomial test followed by Bonferroni post hoc correction. We kept the 7-mer nucleotides as candidate miRNA seed if the conservation signal above background with *P* value < 0.05. (b) Venn plot of known seeds (left circle) from a previous study and detected seeds (right circle). (c) Venn plot of known seeds from eukaryote (left circle) and detected seeds (right circle).

We used an integrated approach to extract candidate miRNA seeds from archaeal genome. The overview of* in silico* detection pipeline is illustrated in Figure 1(a). To start with, we retrieved 3′ UTR information based on the gene annotation in the UCSC. Due to missing 3′-UTR genomic coordinates in the gene annotation of Archaea, here, we defined the 3′-UTR as 80 nucleotides (contain the majority of detected 3′UTR) immediately following the chromosomal coordinates of 3′ terminal of each “Genbank Refseq” gene [12]. Then, we employed a sliding window approach that used continuous 7 nt window with a step size of 1 nt starting from the 5′ terminal of defined 3′-UTR and calculated the “Window Conservation Score” (WCS) by the average conservation score in each window. Sliding windows overlapped with repeat sequence were excluded. Then, all of these WCSs were ranked within each species, and the “Conservation Score of 50th percentile” (CS50) for each species could be readily obtained. In every species, for each type of detected 7-mer nucleotides, we calculated the counts of conservation scores greater or less than the score of CS50, respectively. Then, for each type of 7-mers, we compared these two kinds of counts in all species to check if the difference between them is significant or not. The one-tailed significance binomial test was carried out using PDL::Stats::Basic module by Perl language. We kept the 7-mer nucleotides as candidate miRNA seed if the conservation signal is above background with a *P* value < 0.05 followed by Bonferroni post hoc correction.

The hypergeometric test was used to check if the overlap of two seed sets is significant or not. The probability to find an overlap of *k* seeds between two sets by chance can be given as

(1) $$\begin{array}{c} P_{k}=\frac{C_{M}^{k}C_{N-M}^{n-k}}{C_{N}^{n}}. \end{array}$$

Here, *N* is the number of all types of 7-mer seeds (4^7^ = 16384), *M* is the number of seeds in one of two sets, *n* is the number of seeds in the other set, and *k* is the number of overlap seeds.

To assess functional similarity of miRNA-target genes among species, functional enrichment analysis (functional annotation clustering) was performed on the DAVID Web server [3, 13, 14]. For each species, the target genes were tested against all the genes on the list. In order to decrease the false positive rate, we only used genes linked candidate seed-target region with WCS value greater than the “Conservation Score of 95th percentile” (CS95) in related species for further analysis. Target sites overlapped with coding regions were ruled out. Enrichment analysis was performed for each species with the standard parameters, and a series of functional annotations were selected, which included GOTERM_BP_FAT, GOTERM_CC_FAT, GOTERM_MF_FAT, INTERPRO, PIR_SUPERFAMILY, SMART, BBID, BIOCARTA, KEGG_PATHWAY, COG_ONTOLOGY, SP_PIR_KEYWORDS, UP_SEQ_FEATURE, GENETIC_ASSOCIATION_DB_DISEASE, and OMIM_DISEASE.

## 3. Results and Discussion

### 3.1. Summary of Genome-Wide Seed Prediction

The pipeline for genome-wide seed prediction was outlined in Figure 1(a). After removing some species without conservation information, there were 108 genomes left in our analysis (Supplement Table S1 available online at http://dx.doi.org/10.1155/2014/671059), which belong to two major groups—the Euryarchaeota and the Crenarchaeota. At last, by the sliding window approach, we retrieved 13,779,602 records, including all types (4^7^ = 16384) of 7-mer nucleotides, of which 2649 were remained as candidate seeds with significant signal of conservation information. Our analysis reveals the widespread presence of conserved seed sites in archaeal genome.

The major innovation of our method is taking into account the phylogenetic information that in some way controls for the conservation of miRNA-target interaction locations in defined 3′ UTRs. From defined 3′ UTRs in each considered species, by sliding window approach, we extracted all of 7-mer nucleotides and their corresponding conservation information scores (based on their presence in related archaeal species' genomes), which can be determined by multiple sequence alignment [6, 15]. Because of selective constraint and slower evolving, the region overlapped with miRNA target sites is more likely to be conserved than other region of 3′ UTR, which can be attributed to many factors such as mutation, structure variation, and gene conversion [4, 16]. Comparative genomics has revealed that the conserved miRNA target sites, especially the seed-target interaction sites [2, 5, 17, 18], are present in the 3′ UTRs of coding genes at a considerably higher rate than random ones [1, 16]. Therefore, it is conceivable to predict miRNA seeds from the evolutionary conservation regions.

In UCSC Archaeal Table Browser, the conservation information is calculated from a multiple sequence alignment implemented by several related species, such as a special genus, not by all archaeal genomes (Figure 1(a)). For example, conservation information from two species of two different genera may be determined by different multiple sequence alignments. Therefore, the absolute value of conservation score can only present the phylogenetic information for related species and is not comparable among all of species directly. In order to reduce the effect induced by background conservation scores when comparing among all of species, we used the relative conservation rates that defined high or low conservation through comparing with CS50 in each species. Then, the counts of high and low conservation for each type of 7-mers can be got, respectively. Without selective constraint, the counts of high and low conservation should be equally acquired by chance in every species, and the candidate seed-target sites should be much more conserved than sites predicted by the binomial distribution.

In the seed prediction step, we did not mask any 3′ UTR elements overlapped with “Open Reading Frame” (ORF). Most of these genes are only defined by computer analysis without further experimental validation information. And in our analysis, we used the relative conservation rates, so the target region overlapped with ORF cannot compromise the ability to identify seed-target sites when each 7-mer nucleotide gets the same chance to be located in the coding region. The overlap region usually has more conserved information and is less likely to be changed during the course of evolution, since it suffered pressures from not only the coding selection, but also the conserved seed-target interaction. Therefore, it cannot be discarded in the step of seed-target identification. However, in the following step of predicting target genes function, regions overlapped with ORFs clearly contain greater conservation information than others, and they are excluded to reduce the false positive.

### 3.2. The Effectiveness of Seeds Prediction

To estimate the effectiveness of seeds prediction in pipeline, we compared our candidates with the collected known miRNA seed sets. Twenty-nine unique seeds (nucleotides positioned 2–8) are extracted from the 5′ end of “Most cloned sequences” in previous research [6, 10], of which 11 are detected in our result (Figure 1(b)). The overlap of two sets with significant test score shows that the detected miRNA seeds cannot be assigned by chance.

A large number of miRNA seeds are detected in our analysis, which should be verified by further experiment. Some factors, the poor gene annotation of archaeal genome, for example, limit the sensitivity of our method. Our choice of 7-mer seed is motivated by the observation that shorter seed will produce much noise fragments and increase the false positive [7–9, 19], and longer seed will reduce the number of candidate targets and decrease the power of the statistical test. In addition, our analysis demands perfect seed matching confined to the 3′ UTRs, though some miRNA-target interactions are not constrained by stringent pairings of the seed region and wobbles or mismatches (G : U pairs) are allowed to disrupt seed pairing [10, 20].

### 3.3. Conservation Information of miRNA Seeds

To further explore the phylogenetic information of miRNA seeds between Archaea and eukaryote, we systematically examined a comprehensive list of currently available eukaryotic seeds from the website of TargetScan (http://www.targetscan.org/) [10, 16]. At last, we derived 2470 unique eukaryotic seeds, with or without the family conservation information. Interestingly, Venn plot shows that the overlap of these two seed sets is very significant (*P* value < 1*e* − 8) (Figure 1(c)), which means that the detected seeds are conserved between these two domains of life, suggesting that the miRNA may evolve before the divergence of Archaea and eukaryote.

It is approximately millions of years since the divergence of Archaea and eukaryote [2, 12]. Over this enormous span of time, the accumulation of multiple substitutions in DNA sequence might have erased most of signals that would establish the relationship between archaeal and eukaryotic genes. However, it is suggested that the functional constraints vary across the genome sequence so that sites would evolve with different speed in recent simulations and empirical studies [17, 21–25]. Therefore, low-evolving sites can still retain useful phylogenetic information, explaining why we are able to detect the significant signal of shared miRNA seeds across the two domains of life.

### 3.4. Target Gene Functional Annotation

The existing protocol of miRNAs functional prediction evaluates the strength of miRNA-target interaction by base pair complementarity and hybridization free energy [10], but reliable* in silico* prediction of function has been challenged by the extreme heterogeneity of the genome sequence and the typically short length of 3′-UTR (usually less than 80 nt) [12]. With perfect complementary match and higher conservation than regions with no interaction, the miRNA seed-target region (defined as the nucleotides 2–8 of mature miRNAs) is of critical importance to* in silico* target prediction [17, 22].

**Figure 2:**
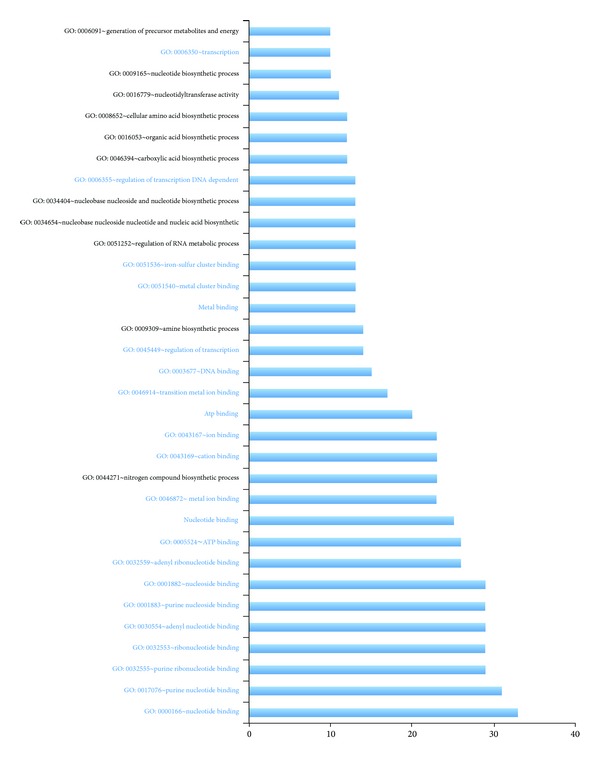
Bar chart shows the functional enrichment analysis results using DAVID. The length of the bar indicates the frequency of terms appearing in the description of functional enrichments (>10). Terms related to transcriptional regulation are indicated in blue.

In order to identify the related function of the candidate seeds, for each species, we defined the corresponding target gene by containing at least one WCS that is greater than the CS95 in the 3′ UTR. The site overlapped with coding region was excluded to decrease the false positive. The proportion of target genes extracted starts from 0.01% to 1.6%, with the average of 0.16% (Supplemental Table S2). The “Id Confusion” occurred when we submitted our gene lists to the DAVID service, and 38 species were approved for further enrichment analysis. The proportion of predicted miRNA target genes falling in each of functional annotations can be discovered in Supplementary Table S3. The bar chart is used to show the top frequency (≥10) of the target gene annotation descriptions in enrichment analysis. We found that the miRNA targets are enriched for genes involved in transcriptional regulation such as nucleotide binding and ion binding (Figure 2), which coincides with previous conclusion in eukaryote [11, 22], supporting the hypothesis that miRNA may evolve before the divergence of two domains of cellular life.

Notably, various target genes are predicted with function related to the metabolic and biosynthesis process (Figure 2). It is reasonable that under extreme condition Archaea need to resist the biosynthesis process because of poor material for the cellular building formation. For instance,* Nanoarchaeum equitans *lacks most of the genes for metabolic and biosynthesis process [12, 26]. Other related functions show that the regulation of archaeal miRNA genes has a very broad diversity of biological processes.

## 4. Conclusions

Here, we introduced a computational approach using phylogenetic diversity information from miRNA seed-target evolution, to predict miRNA seeds through the identification of their matches at conserved positions within defined 3′ UTRs. Despite the presence of false positives, evidence that the detected miRNA seeds are supported by the previous study indicates that our prediction is still powerful. The overlap of two seed sets from Archaea and eukaryote with significant signal gives us the clue that the miRNA may evolve before the divergence of these two domains of cellular life. The evidence that enriched predicted targets are enriched for genes involved in transcriptional regulation is also consistent with this hypothesis.

## Supplementary Material

Supplementary S1: List of candidate seeds that were extracted from 115 archaeal genomes. The conservation signal is above background with a P-value <0.05 followed by Bonferroni post hoc correction.
Supplementary S2: List of miRNA target sites that were detected with ‘Window Conservation Score' value greater than the ‘Conservation Score of 95th percentile' in related species. The sites overlapped with coding regions were discarded. See the Material and Methods section for more details.
Supplementary S3: Detail information of the functional enrichment analysis results using DAVID. There are 38 species approved in this analysis. These tables are generated from the DAVID analysis, and we simply integrate them together using the original description. The field definitions are added on the top of each table to make it more clear. The type of GO term or KEGG pathways can be found in the Category column.
